# Asymptotic Properties of Pearson's Rank-Variate Correlation Coefficient under Contaminated Gaussian Model

**DOI:** 10.1371/journal.pone.0112215

**Published:** 2014-11-13

**Authors:** Rubao Ma, Weichao Xu, Yun Zhang, Zhongfu Ye

**Affiliations:** 1 Department of Automatic Control, School of Automation, Guangdong University of Technology, Guangzhou, Guangdong, China; 2 Department of Electronic Engineering and Information Science, University of Science and Technology of China, Hefei, Anhui, China; Beijing University, China

## Abstract

This paper investigates the robustness properties of Pearson's rank-variate correlation coefficient (PRVCC) in scenarios where one channel is corrupted by impulsive noise and the other is impulsive noise-free. As shown in our previous work, these scenarios that frequently encountered in radar and/or sonar, can be well emulated by a particular bivariate contaminated Gaussian model (CGM). Under this CGM, we establish the asymptotic closed forms of the expectation and variance of PRVCC by means of the well known Delta method. To gain a deeper understanding, we also compare PRVCC with two other classical correlation coefficients, i.e., Spearman's rho (SR) and Kendall's tau (KT), in terms of the root mean squared error (RMSE). Monte Carlo simulations not only verify our theoretical findings, but also reveal the advantage of PRVCC by an example of estimating the time delay in the particular impulsive noise environment.

## Introduction

Correlation coefficients are indices that depict the strength of statistical relationship between two random variables obeying a joint probability distribution [1]. In general, correlation coefficients should be large and positive if there is a high probability that large (small) values of one variable occur in conjunction with large (small) values of another; and it should be large and negative if the direction reverses [2]. Due to their theoretical and algorithmic advantages, correlation coefficients have been widely used in many sub-areas of signal processing [3]–[18]. Among many methods of correlation analysis in practice, Pearson's product moment correlation coefficient (PPMCC), Kendall's tau (KT) and Spearman's rho (SR) are perhaps the most prevalent ones [19].

There are many advantages and disadvantages to these three classical coefficients. PPMCC is optimal under the bivariate normal model (BNM), and is appropriate mainly for characterizing linear correlations. However, it will output misleading results if nonlinearity is involved in the data. On the other hand, the two rank-based coefficients, SR and KT, are not as powerful as PPMCC when the data follows bivariate normal distributions. Nevertheless, they are invariant under increasing monotone transformations, which makes them more suitable for many nonlinear cases in practice. Moreover, theoretical and empirical results indicate that SR and KT surpass PPMCC when data are corrupted by impulsive noise [12], [20]. Besides these three classical coefficients, some methods such as Pearson's rank-variate correlation coefficient (PRVCC) [21], Gini correlation (GC) [22], and order statistics correlation coefficient (OSCC) [23]–[25] among others [26]–[29], have also been proposed in the literature.

It was known long before that PPMCC is an optimal estimator of the population correlation coefficient, in the sense of unbiasedness and approaching the Cramer-Rao lower bound for large samples under bivariate normal models [30]. Despite its desired properties just mentioned, PPMCC might not be applicable under the circumstances where the data are corrupted by impulsive noise, that is, the distribution of data deviates from the BNM. Consider the following scenario that frequently occurs in radar, sonar or communication. We have a prescribed signal, whether deterministic or stochastic, whose statistical property is priorly known. Our purpose is to estimate the correlation between this “clean” signal and the associated distorted version from the receiver that might be corrupted by a tiny fraction of impulsive noise (outliers with very large variance [31]–[33]). To deal with such case, one might adopt the conventional strategy, that is, ranking the cardinal variable(s) and resorting afterwards to SR or KT [22], which are robust against both nonlinearity and impulsive noise [20]. However, using only ranks of the two variables, we unavoidably lose useful information embedded in the variates of the “clean” variable. A better strategy would be to rely on coefficients, such as PRVCC [21], that accommodate both ordinal and cardinal information contained in the samples. Our purpose in this work is thus to investigate the properties of the historical PRVCC by both theoretical and empirical means.

The contribution in this work is twofold. Firstly, we establish the asymptotic closed forms of the expectation and variance of PRVCC under a contaminated Gaussian model that emulates a frequently encountered scenario in practice. Secondly, we demonstrate the superiority of PRVCC over PPMCC, SR and KT, in terms of the root mean squared error, by an example of estimating the time delay in the particular impulsive noise environment. These theoretical and empirical findings might be helpful to rejuvenate the historical PRVCC, which has long been forgotten in the literature due to insufficient understanding on its theoretical properties.

For convenience of later discussion, we employ symbols 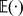, 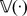, 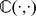 and 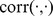 to denote the mean, variance, covariance and correlation of (between) random variables, respectively. Univariate and bivariate normal distributions are denoted by 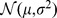 and 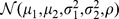, respectively. The sign 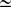 reads “is approximately equal to”, whereas the sign 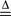 stands for “is defined as”. The notation 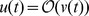 denotes that 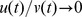 as 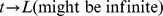 [34]. The symbol 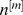 stands for the product 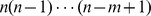. Other notations will be defined where it first enters the text.

## Methods

This section presents the definitions of PRVCC as well as a particular CGM model simulating the impulsive noise environment mentioned in the previous section. Moreover, some auxiliary results are also established for further theoretical analysis.

### 1 Definitions of PRVCC

Let 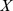 and 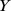 be two random variables following a continuous bivariate distribution. Denote by 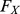 and 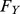 the marginal distributions of 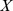 and 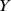, respectively. Then, according to a historical paper of Pearson [21], one of the population versions of PRVCC can be defined, in modern notation, by
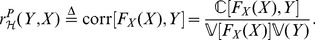
(1)Exchanging the roles of 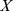 and 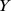 in (1) yields the other version
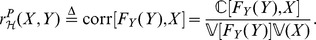
Let 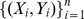 be 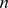 independent and identically distributed (i.i.d.) data pairs drawn from a continuous bivariate population. After rearranging 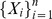 in ascending order, we get a new sequence 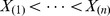, which is termed the order statistics of 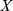 [35]–[37]. Suppose that 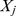 is at the 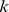
th position in the sorted sequence. The integer 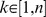 is named the rank of 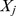 and is denoted by 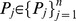. Let 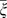 represent the arithmetic mean of 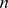 data points 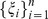. Then, based on (1), the sample version with respect to 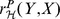 can be constructed as
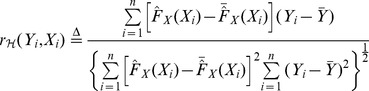
(2)where 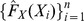 is the empirical cdf of 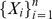. Substituting the relationship 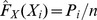 into (2) along with some simplifications leads to
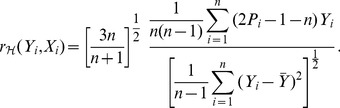
(3)Exchanging the role of 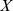 and 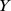 in (3) gives another sample version 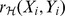 with respect to 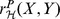. Note that in general 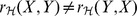. The choice between 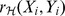 and 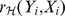 depends on different roles played by 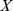 and 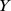 in the scenario mentioned in the previous section. To avoid redundancy, we will focus on the properties of 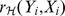 which is abbreviated as 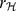 in the sequel unless ambiguity occurs.

### 2 Contaminated Gaussian Model

To simulate the specific circumstance remarked in Section **Introduction**, throughout we utilize the following CGM representing the joint probability density function (pdf) of two random variables 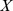 and 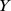 [20]

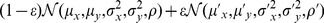
(4)where 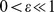, 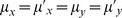, 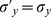, 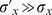 and 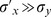. Under this specific CGM, it is obvious that the marginal distribution of 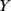 is 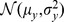, whereas the marginal distribution of 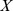 is 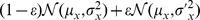. In other words, under Model (4), 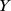 stands for a “clean” normal variable while 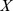 stands for a “dirty” variable corrupted by a tiny fraction of Gaussian component with vary large variance (might tending to infinity). In this model, the parameter 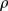, which is considered of interest, is what we aim at estimating as accurate as possible, while 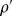 and 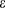 are interferences we seek to suppress. For the reason why Model (4) can happen in practice, please see Appendix A in our previous work [20].

### 3 Auxiliary Results

To establish our major results of Theorem 1 in the next subsection, some auxiliary results summarized in Lemma 1 below are mandatory.

#### Lemma 1

*Assume that the random vector* 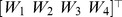 *follows a quadrivariate normal distribution with* 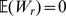 *,* 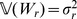 *, and* 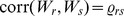 *for* 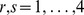 *. Write* 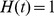 *for* 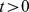 *and* 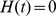 *for* 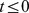 *. Then*

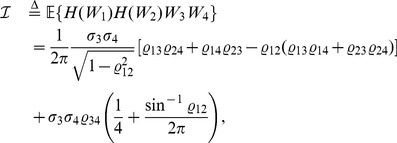
(5)




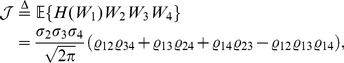
(6)




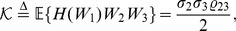
(7)
*and*

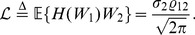
(8)
*Proof.* Write 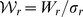 for 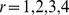. Then
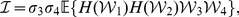
(9)




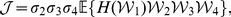
(10)




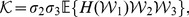
(11)




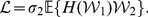
(12)The results of (5) and (7) follow readily by substituting the results in [39] into the right sides of (9) and (11), respectively. Next we show that (6) and (8) also hold true. Let 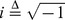. Then, according to [39], it follows that
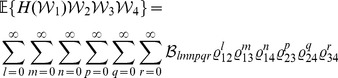
(13)where
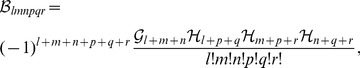
(14)

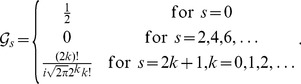
(15)and
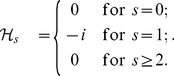
(16)It is seen that 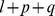, 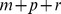 and 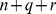 are all subscripts of the 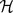-terms in (14). Since, by (16), only 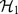 is non-null, then (14) and hence (13) are non-null only when the following conditions are satisfied, i.e.,
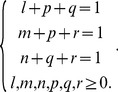
(17)It is easy to verify that there are only four solutions to (17), as
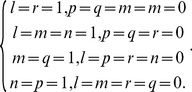
(18)Substituting (18) into (13) and using (15) and (16) thereafter produce
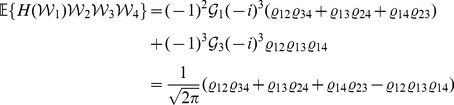
(19)which along with (10) leads to (6). By a similar argument, we have
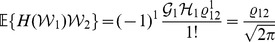
(20)which together with (12) yields (8). This completes the proof of the lemma.□

### 4 Asymptotic Mean and Variance of PRVCC Under CGM (4)

By applying the *delta method* [38] with Lemma 1, we are ready to establish the closed forms of the mean and variance of PRVCC for samples generated by CGM (4).

#### Theorem 1

*Let* 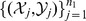 *be* 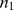 *i.i.d. data pairs drawn from a bivariate normal population* 
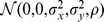
 *and* 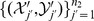 *be* 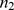 *i.i.d. data pairs drawn from another bivariate normal population* 
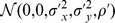
 *. Assume that* 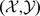 *and* 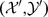 *are mutually independent. Write* 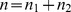 *and* 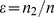 *. Denote by* 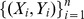 *the union of* 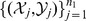 *and* 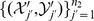 *. Then, as* 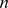 *large,* 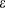 *small,* 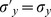 *,* 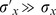 *and* 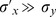 *, the expectation and variance of PRVCC defined in (3) are*

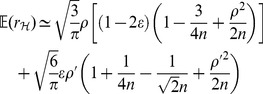
(21)
*and*

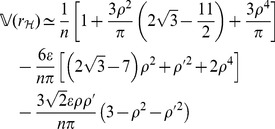
(22)
*Proof.* From (3), it is easy to verify that 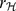 is shift invariant. Therefore, we lose no generality by assuming that 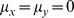 hereafter. For convenience, write
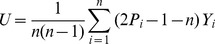
(23)




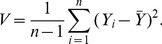
(24)Then, by the well known *delta method* [38], it follows that
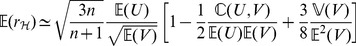
(25)and
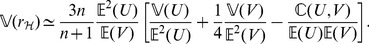
(26)Obviously, we only need to evaluate 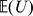, 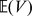, 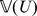, 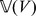 and 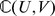 in order to work out (25) and (26). From the theorem assumption, both 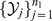 and 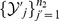 follow the same normal distribution 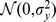, then 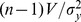 obeys a 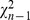 distribution, which means that
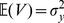
(27)




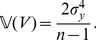
(28)Using Lemma 1 with some tedious algebra, we can obtain 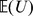, 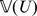 and 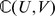.

We first derive 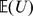. From the definition (23) and the relationships 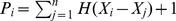 [40],
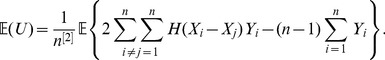
(29)Expanding and recalling that 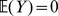, it follows that
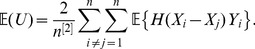
(30)Since, by definition, 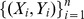 is a mixture of 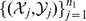 and 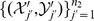, (30) can be expanded as
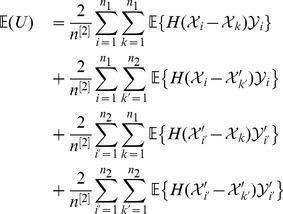
(31)which becomes (32) after some straightforward algebra along with the assistance of (8) in Lemma 1.
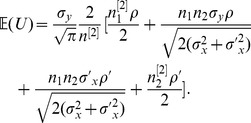
(32)


Next we evaluate 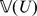, which can be written as
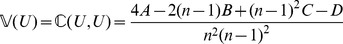
(33)where
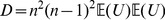
(34)




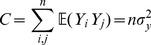
(35)




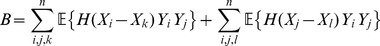
(36)and
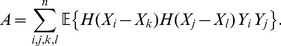
(37)The expression of 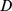 is easily obtained by substituting (31) into (34).

For convenience, denote by 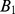 and 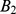 the two triple summations of 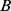 in (36). Then it follows that 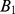 is decomposable into eight sub-triple summations which can be further partitioned into 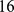 disjoint and exhaustive subsets that listed in Table 1. An application of (7) to Table 1 leads directly to
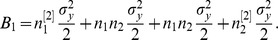



**Table 1:** Quantities for Evaluation of 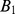 in Eq. (36).

Subsets	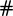 of terms	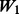	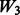	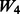	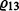	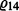	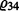
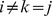	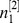	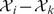	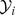	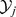	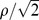	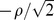	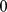
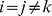	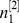	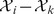	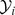	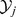	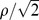	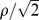	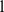
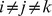	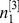	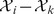	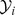	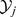	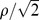	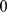	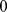
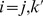	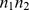	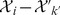	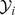	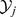	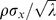	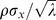	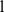
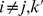	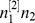	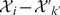	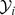	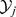	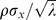	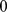	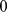
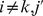	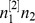	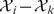	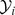	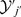	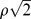	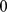	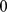
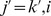	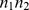	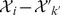	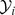	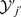	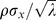	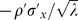	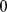
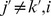	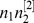	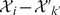	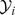	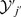	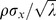	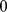	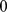
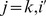	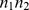	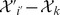	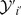	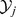	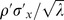	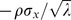	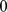
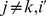	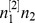	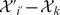	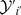	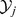	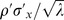	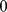	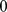
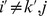	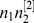	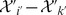	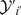	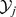	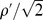	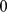	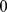
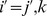	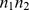	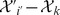	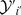	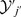	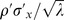	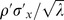	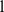
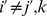	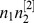	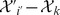	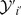	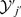	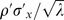	0	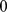
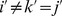	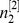	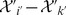	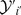	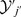	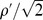	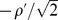	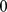
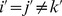	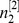	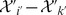	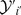	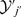	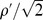	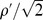	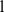
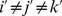	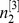	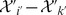	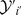	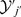	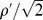	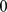	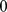

1 In the second column 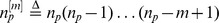 for 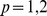.

2 In the columns for 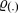 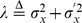.

Similarly we also have
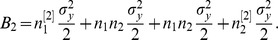
Thus
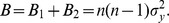
The quadruple summation in (37) can be decomposed as
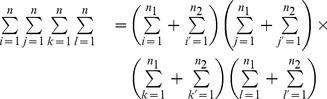
(38)Expanding (38) according to different suffixes of 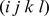 and 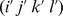, we obtain 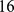 sub-quadruple summations which can be further partitioned into 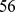 disjoint and exhaustive subsets. In other words, 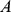 is a summation of 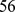 integrals of the form 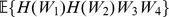, i.e., the 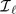-terms, weighted by corresponding subset cardinality, i.e., the 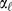-terms. By substituting into (5) the corresponding parameters tabulated in Table 2 as well as exploiting the symmetry of (38), we obtain the expression of 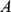. Substituting the expressions of 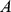, 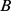, 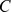 and 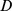 into (33) and tidying up lead to the expression of 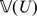 in (39).
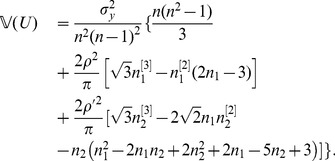
(39)


**Table 2:** Quantities for Evaluating 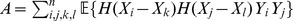 in Eq. (37).

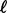	Subsets	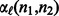	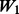	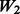	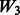	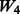	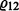	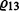	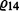	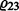	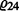	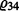
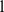	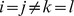	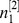	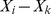	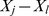	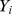	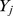	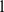	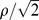	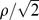	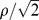	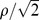	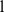
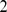	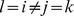	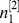	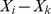	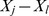	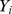	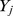	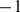	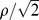	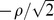	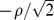	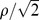	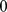
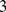	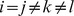	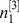	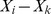	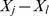	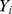	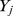	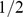	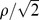	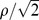	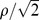	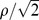	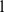
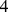	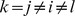	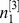	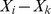	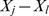	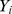	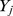	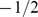	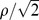	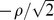	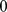	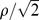	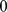
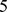	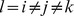	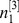	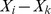	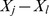	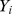	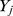	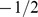	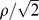	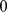	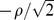	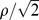	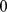
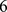	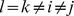	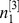	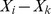	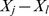	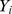	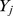	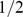	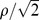	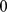	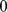	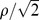	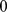
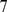	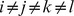	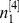	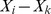	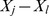	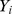	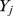	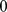	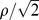	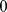	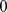	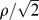	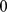
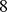	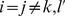	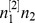	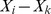	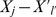	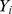	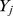	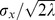	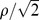	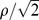	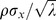	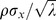	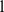
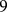	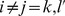	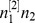	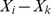	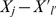	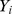	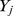	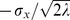	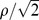	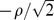	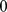	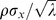	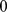
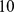	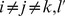	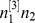	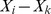	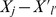	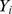	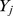	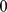	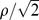	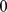	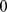	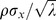	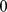
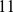	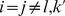	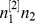	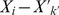	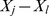	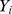	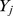	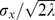	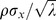	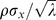	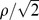	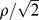	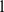
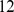	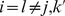	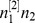	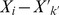	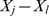	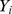	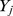	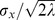	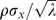	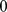	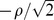	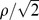	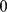
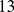	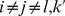	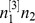	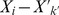	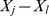	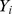	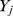	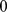	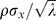	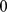	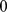	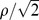	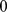
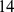	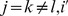	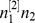	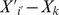	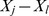	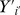	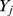	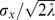	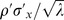	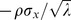	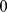	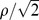	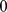
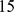	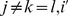	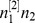	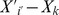	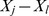	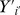	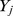	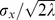	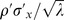	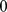	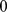	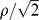	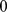
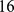	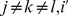	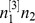	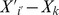	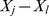	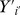	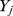	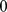	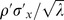	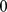	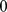	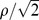	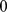
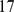	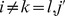	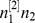	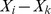	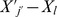	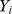	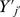	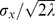	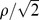	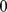	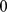	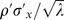	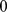
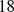	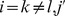	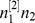	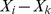	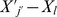	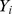	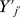	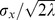	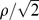	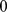	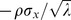	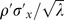	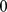
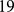	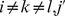	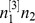	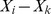	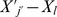	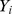	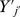	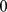	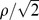	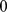	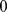	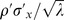	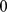
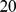	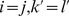	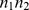	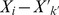	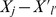	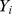	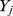	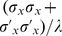	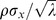	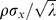	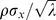	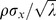	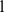
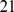	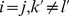	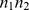	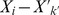	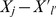	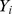	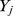	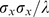	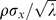	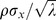	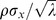	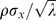	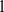
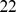	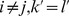	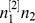	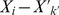	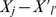	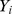	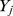	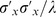	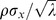	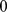	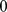	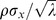	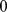
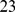	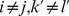	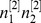	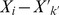	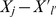	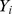	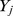	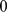	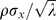	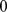	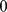	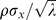	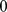
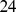	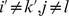	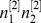	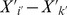	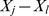	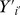	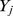	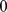	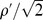	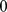	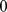	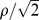	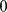
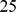	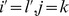	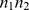	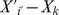	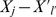	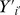	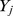	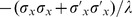	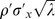	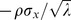	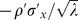	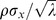	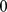
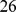	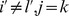	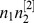	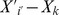	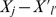	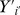	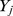	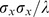	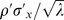	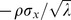	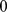	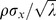	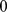
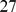	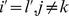	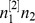	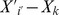	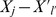	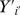	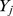	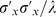	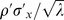	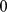	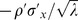	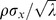	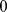
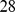	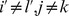	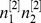	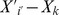	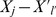	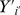	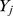	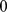	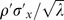	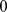	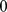	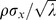	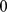

1 In the third column 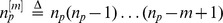 for 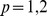.

2 In the columns for 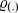 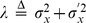.

Finally we deal with 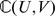. Write
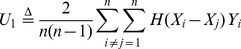
(40)and
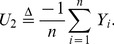
(41)Then 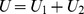. Now
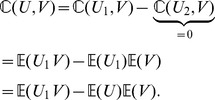
(42)Since we have 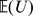 in (32) and 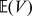 in (27), the second term in (42) can be easily obtained, as
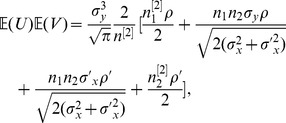
(43)whereas the first term, 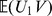, can be written as
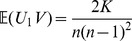
(44)where
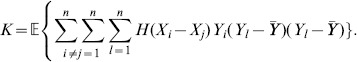
(45)It is evident that 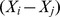, 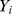, 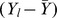 and 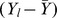 can be regarded as 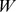-terms in Lemma 1, i.e.,
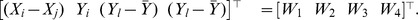
As mentioned above, 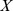 is a union of 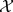 and 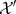, and 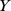 is a union of 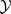 and 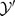, the triple summation in (45) can be split into eight terms as
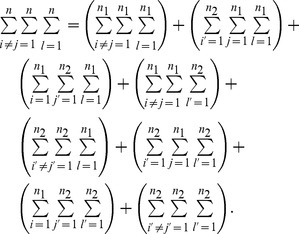
(46)Using (6) in Lemma 1 along with the corresponding parameters tabulated in Table 3, we can work out each sub-triple summation in (46). A series of straightforward algebra leads readily to (47).
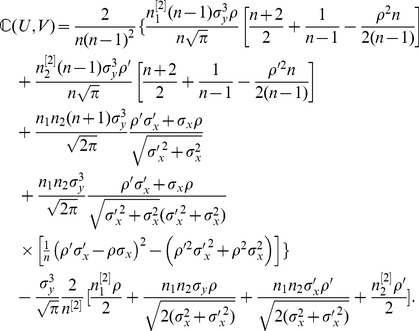
(47)


**Table 3:** Quantities for Evaluation of 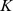 in Eq. (45).

Subsets	#  of terms	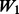	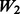	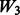	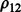	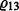	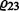
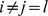	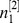	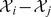	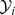	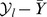	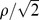	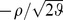	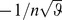
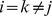	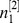	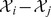	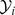	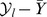	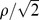	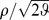	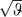
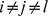	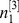	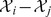	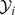	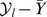	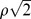	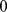	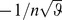
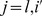	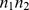	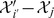	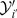	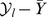	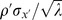	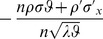	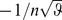
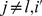	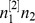	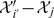	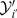	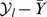	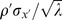	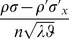	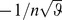
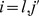	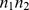	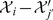	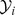	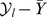	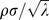	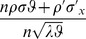	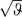
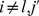	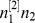	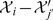	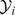	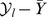	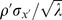	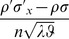	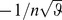
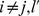	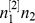	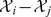	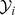	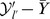	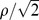	0	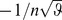
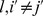	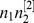	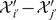	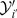	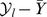	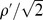	0	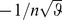
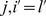	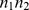	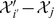	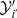	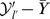	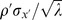	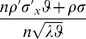	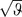
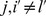	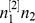	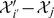	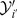	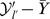	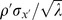	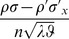	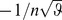
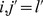	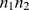	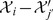	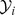	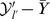	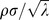	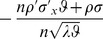	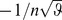
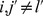	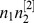	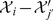	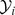	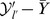	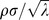	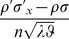	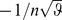
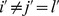	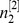	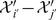	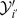	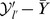	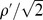	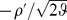	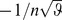
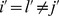	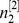	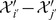	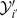	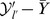	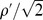	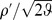	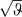
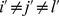	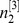	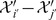	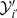	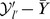	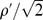	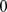	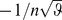

1 In the second column 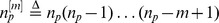 for 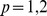.

2 In the columns for 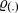 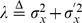 and 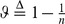.

Substituting (27),(28),(32),(39) and (47) into (25) and (26), respectively, letting 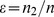 and 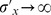, and omitting 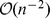 terms thereafter, we finally arrive at (21) and (22), respectively. The theorem thus follows.□

#### Remark 1

*Letting* 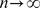 *in (21),* 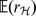 *simplifies to a neater form of*

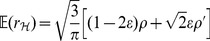
(48)
*which manifests how* 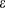 *and* 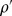 *affect the accuracy of the estimate of* 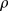 *by PRVCC. Moreover, as* 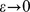 *, (21) and (22) reduce respectively to*

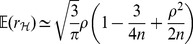
(49)
*and*

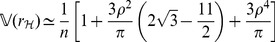
(50)
*which are the contamination-free versions of PRVCC.*

## Results and Discussion

In this section we verify the correctness of Theorem 1 by Monte Carlo simulations. To gain a further insight about PRVCC, we also compare it with the two classical correlation coefficients,i.e., SR and KT, in terms of RMSE. At last, we will provide an examples of time-delay estimation under CGM (4). Since the theoretical results in Theorem 1 only hold true for large sample size 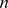 and small 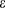, in this section we set the sample size 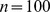 and 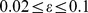. All samples are generated by suitable functions in the Matlab environment. For the sake of accuracy, the number of Monte Carlo trials is set to be 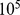 unless otherwise stated.

### 1 Verification of Theorem 1

**Figure 1:**
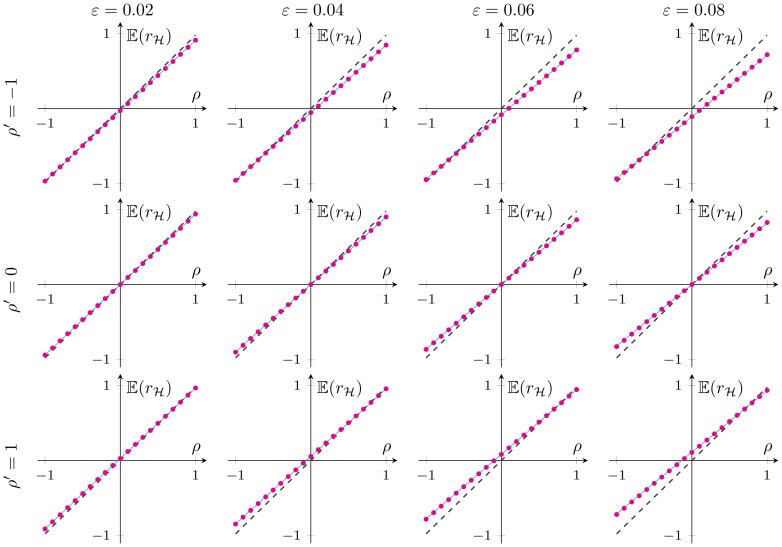
The numerical verification of (21), the expectation of 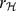 in Theorem 1. The number of samples is chose as 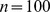. In the vertically up direction, 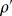 is decreasing following 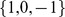 respectively; whereas 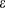 corresponds to a increasing trend in the horizontally right direction, following 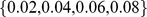 respectively. It shows a good agreement between the simulation result (circles) and the theoretical computation (solid lines) in each subplot. As a reference, the contamination-free version (49) is also posted together (see dashed curves).

Figure 1. verifies the correctness of the mean of PRVCC under CGM (4) for large samples and small 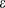. Specifically, in Figure 1. we plot the simulation results (circles) and the theoretical results of (21) (solid lines), and the contamination-free version (49) (dashed lines) under different combinations of 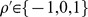 and 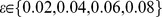. Good agreements are observed between the simulation results and the theoretical counterparts. It can also be observed that the larger the contamination fraction 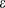 and difference between 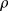 and 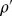, the bigger the bias between 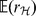 and the ideal dashed curve corresponding to 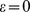.

**Figure 2:**
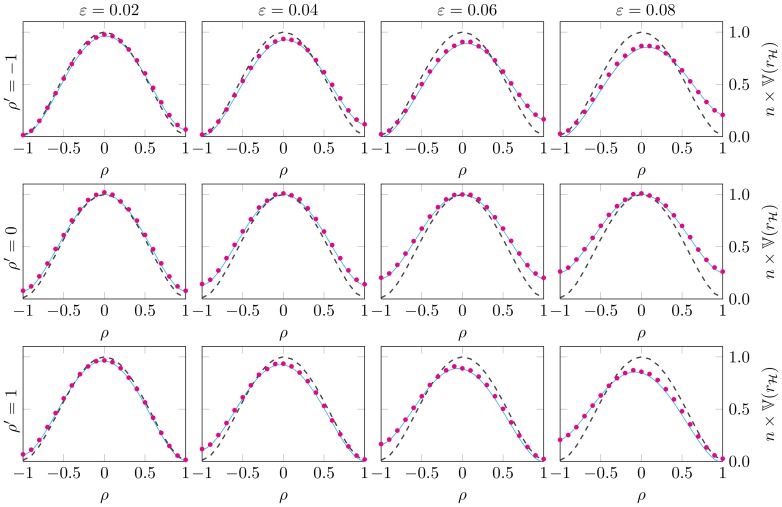
The numerical verification of (22), the variance of 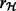 in Theorem 1. The number of samples is chose as 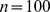. In the vertically up direction, 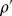 is decreasing following 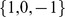 respectively; whereas 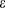 corresponds to a increasing trend in the horizontally right direction, following 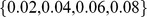 respectively. It shows a good agreement between the simulation result (circles) and the theoretical computation (solid lines) in each subplot. As a reference, the contamination-free version (50) is also posted together (see dashed curves).

Figure 2. verifies the correctness of the variance of PRVCC, by plotting the simulation results (circles) and the theoretical results of (22) (solid lines) concerning 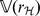 in the same scenarios as in Figure 1. For the purpose of comparison, the contamination-free version (50) (dashed lines) is also included in each subplot to highlight the effects of 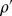 and 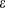. Note that we have multiplied 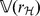 by 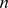 for a better visual effect. This figure shows good agreements between the simulation results and the corresponding theoretical ones. Moreover, it is seen that when 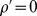, the curves are symmetric and the magnitude of 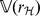 increase with 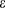, especially for 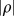 large. On the other hand, when 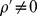, the curves are no longer asymmetric. Specifically, for 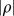 large, 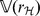 increases if 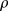 and 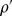 have opposite signs; and it decreases if 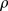 and 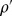 have the same signs. When 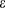 is fixed, 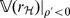 is the reversal of 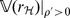.

From these two figures, it follows that, although derived based on the assumptions 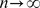 and 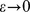, our theoretical results established in Theorem 1 are sufficiently accurate for 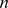 as small as 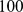 and 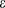 as large as 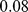. This means that, PRVCC is applicable to many situations in practice, such as radar and biomedical engineering, where the sample size 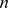 is much larger than 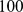 and the fraction of impulsive interference 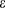 is much lower than 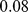.

### 2 RMSE Comparison of PRVCC with SR amd KT

To deepen the understanding of PRVCC, in this subsection we compare in terms of RMSE the performance of PRVCC with SR and KT, which are also robust under the CGM (4) as shown in our previous work [20].

For fairness of comparison, some calibrations are necessary. From (21), it follows
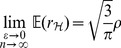
(51)by which we can define an asymptotic unbiased estimator of the population correlation 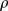, as
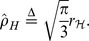
The other two asymptotic unbiased estimators based on KT and SR are defined as [20]

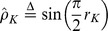





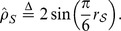
Given definitions of the 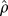 above, we can then compare their performance in terms of the popular RMSE defined by
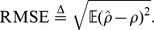
The CGM based on (4) is set to be
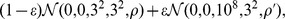
where 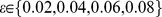, 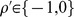 and 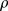 increases from 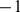 to 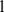 by a step of 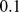. The RMSEs are listed in Table 4, where the minima with respect to 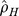, 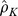 and 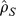 are highlighted in bold font in a rowwise manner within each of the eight blocks. It appears that 1) all RMSEs are quite small, meaning that these three estimators perform similarly well under CGM (4); 2) 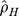 outperforms 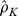 and 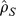 in the cases that 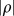 takes values from 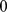 to medium magnitudes; 3) 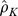 outperforms 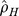 for 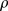 around 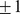; 4) 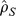 plays an intermediate role between 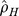 and 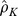. Note that the RMSE values for 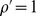 are not shown in Table 4 due to symmetry.

**Table 4:** RMSE of Three Estimators for 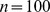, 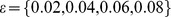 and 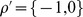.

	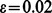			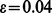			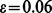			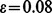		
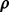	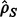	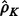	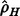	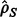	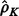	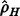	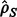	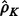	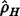	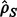	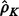	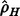
−1.0	0.0111	**0.0007**	0.0187	0.0200	**0.0023**	0.0287	0.0287	**0.0047**	0.0389	0.0369	**0.0078**	0.0488
−0.9	0.0254	**0.0233**	0.0262	0.0278	**0.0257**	0.0301	0.0306	**0.0287**	0.0346	0.0336	**0.0320**	0.0396
−0.8	0.0416	0.0402	**0.0400**	0.0411	**0.0402**	**0.0402**	0.0404	**0.0401**	0.0404	**0.0394**	0.0396	0.0405
−0.7	0.0563	0.0551	**0.0537**	0.0548	0.0538	**0.0525**	0.0532	0.0523	**0.0510**	0.0519	0.0508	**0.0498**
−0.6	0.0687	0.0678	**0.0659**	0.0670	0.0659	**0.0641**	0.0666	0.0652	**0.0635**	0.0665	0.0647	**0.0627**
−0.5	0.0793	0.0788	**0.0765**	0.0787	0.0779	**0.0757**	0.0797	0.0784	**0.0760**	0.0820	0.0803	**0.0772**
−0.4	0.0882	0.0881	**0.0856**	0.0889	0.0884	**0.0858**	0.0918	0.0910	**0.0876**	0.0972	0.0962	**0.0916**
−0.3	0.0950	0.0952	**0.0924**	0.0977	0.0978	**0.0943**	0.1037	0.1037	**0.0991**	0.1123	0.1124	**0.1058**
−0.2	0.1013	0.1018	**0.0989**	0.1057	0.1063	**0.1022**	0.1146	0.1154	**0.1094**	0.1269	0.1283	**0.1197**
−0.1	0.1051	0.1059	**0.1026**	0.1125	0.1134	**0.1087**	0.1251	0.1266	**0.1194**	0.1412	0.1436	**0.1331**
0.0	0.1074	0.1083	**0.1050**	0.1173	0.1185	**0.1133**	0.1345	0.1364	**0.1282**	0.1543	0.1573	**0.1456**
0.1	0.1085	0.1093	**0.1057**	0.1226	0.1236	**0.1181**	0.1431	0.1449	**0.1362**	0.1672	0.1702	**0.1579**
0.2	0.1082	0.1086	**0.1050**	0.1263	0.1267	**0.1213**	0.1509	0.1521	**0.1436**	0.1809	0.1832	**0.1712**
0.3	0.1058	0.1056	**0.1025**	0.1287	0.1281	**0.1236**	0.1583	0.1579	**0.1510**	0.1925	0.1930	**0.1828**
0.4	0.1021	0.1008	**0.0984**	0.1303	0.1276	**0.1251**	0.1658	0.1625	**0.1585**	0.2044	0.2015	**0.1951**
0.5	0.0979	0.0949	**0.0942**	0.1320	**0.1260**	0.1270	0.1723	**0.1645**	0.1660	0.2152	0.2070	**0.2069**
0.6	0.0924	**0.0869**	0.0890	0.1321	**0.1212**	0.1282	0.1783	**0.1639**	0.1731	0.2259	**0.2095**	0.2193
0.7	0.0860	**0.0764**	0.0837	0.1323	**0.1138**	0.1300	0.1831	**0.1584**	0.1801	0.2355	**0.2068**	0.2315
0.8	0.0789	**0.0628**	0.0784	0.1322	**0.1020**	0.1326	0.1879	**0.1475**	0.1882	0.2443	**0.1967**	0.2440
0.9	0.0726	**0.0450**	0.0750	0.1320	**0.0825**	0.1361	0.1925	**0.1259**	0.1970	0.2528	**0.1733**	0.2575
1.0	0.0660	**0.0047**	0.0752	0.1296	**0.0179**	0.1414	0.1933	**0.0391**	0.2068	0.2567	**0.0679**	0.2708
−1.0	0.0432	**0.0028**	0.0511	0.0795	**0.0095**	0.0898	0.1153	**0.0195**	0.1279	0.1508	**0.0328**	0.1652
−0.9	0.0509	**0.0342**	0.0529	0.0819	**0.0531**	0.0858	0.1133	**0.0748**	0.1189	0.1454	**0.0985**	0.1524
−0.8	0.0594	**0.0504**	0.0587	0.0840	**0.0678**	0.0849	0.1106	**0.0883**	0.1130	0.1376	**0.1106**	0.1411
−0.7	0.0696	**0.0644**	0.0673	0.0876	**0.0783**	0.0864	0.1086	**0.0956**	0.1085	0.1309	**0.1150**	0.1318
−0.6	0.0788	0.0760	**0.0759**	0.0915	**0.0863**	0.0892	0.1072	**0.1000**	0.1055	0.1243	**0.1153**	0.1234
−0.5	0.0867	0.0852	**0.0837**	0.0954	**0.0927**	**0.0927**	0.1063	**0.1025**	0.1040	0.1187	**0.1139**	0.1169
−0.4	0.0932	0.0927	**0.0902**	0.0985	0.0974	**0.0959**	0.1060	0.1044	**0.1035**	0.1139	0.1117	**0.1116**
−0.3	0.0985	0.0986	**0.0958**	0.1014	0.1013	**0.0988**	0.1060	0.1056	**0.1033**	0.1102	0.1096	**0.1078**
−0.2	0.1022	0.1027	**0.0996**	0.1038	0.1043	**0.1013**	0.1054	0.1058	**0.1029**	0.1072	0.1075	**0.1048**
−0.1	0.1043	0.1052	**0.1021**	0.1048	0.1055	**0.1024**	0.1049	0.1056	**0.1027**	0.1056	0.1063	**0.1033**
0.0	0.1053	0.1061	**0.1030**	0.1052	0.1061	**0.1029**	0.1050	0.1059	**0.1027**	0.1050	0.1058	**0.1028**
0.1	0.1044	0.1052	**0.1020**	0.1051	0.1059	**0.1028**	0.1051	0.1058	**0.1028**	0.1055	0.1062	**0.1032**
0.2	0.1023	0.1029	**0.0999**	0.1035	0.1039	**0.1010**	0.1050	0.1053	**0.1026**	0.1072	0.1075	**0.1048**
0.3	0.0984	0.0986	**0.0957**	0.1013	0.1012	**0.0987**	0.1055	0.1052	**0.1029**	0.1106	0.1100	**0.1081**
0.4	0.0930	0.0924	**0.0900**	0.0989	0.0979	**0.0962**	0.1054	0.1038	**0.1029**	0.1140	**0.1118**	0.1119
0.5	0.0865	0.0850	**0.0835**	0.0956	0.0930	**0.0929**	0.1059	**0.1022**	0.1036	0.1187	**0.1139**	0.1167
0.6	0.0788	**0.0759**	**0.0759**	0.0916	**0.0864**	0.0894	0.1069	**0.0997**	0.1055	0.1244	**0.1154**	0.1237
0.7	0.0693	**0.0642**	0.0671	0.0876	**0.0784**	0.0865	0.1083	**0.0953**	0.1083	0.1305	**0.1147**	0.1314
0.8	0.0597	**0.0506**	0.0589	0.0843	**0.0680**	0.0853	0.1109	**0.0888**	0.1131	0.1376	**0.1105**	0.1409
0.9	0.0506	**0.0341**	0.0526	0.0817	**0.0531**	0.0856	0.1136	**0.0749**	0.1192	0.1453	**0.0983**	0.1524
1.0	0.0430	**0.0028**	0.0508	0.0792	**0.0094**	0.0896	0.1155	**0.0196**	0.1281	0.1506	**0.0328**	0.1652

In the upper panel are RMSEs for 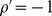; whereas in the lower panel are RMSEs for 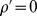. In each of the eight blocks, the minima of RMSE with respect to 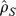, 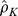 and 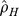 are highlighted in bold font in a rowwise manner.

### 3 Example of Time-Delay Estimation

**Figure 3:**
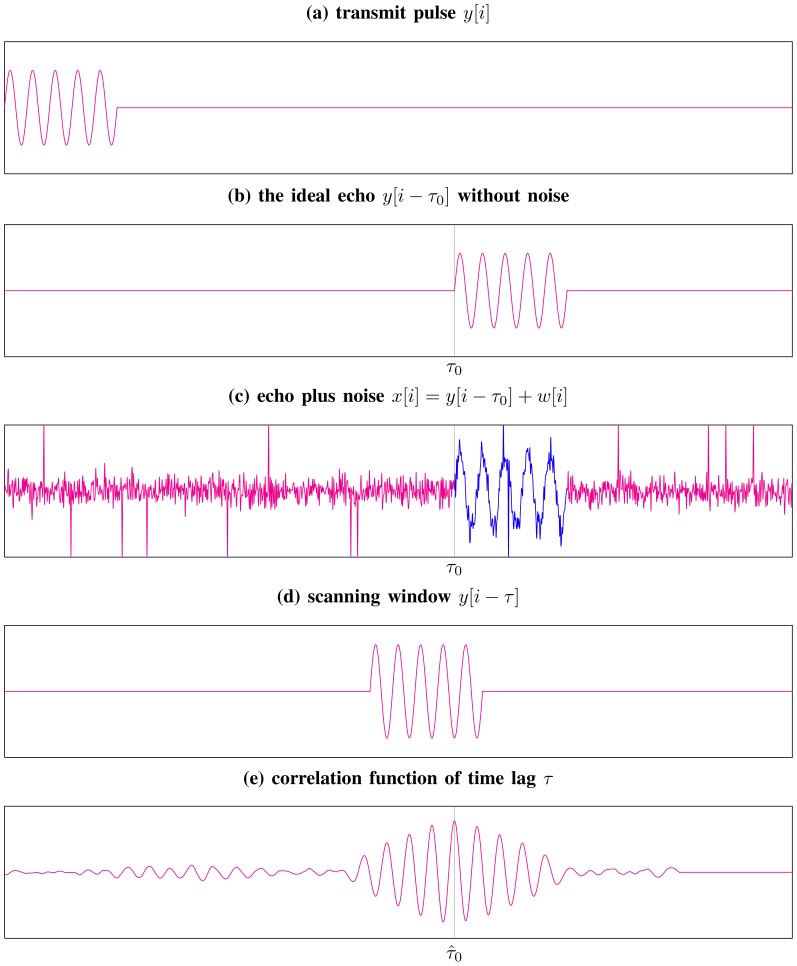
Schematic illustration of estimating the time-delay 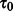 in Model (4). The time-shift 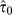 with respect to the maximum of the correlation function in the bottom panel is considered as an estimate of the true time-delay 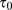.

As remarked in Section **Introduction**, it is often encountered in radar, sonar or communication that we need to estimate the correlation between a prescribed “clean” signal with a distorted version corrupted by impulsive noise. Now we provide an example of time-delay estimation which is similar to this situation. In this example, the prescribed clean signal is a segment of sinusoidal wave
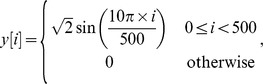
whereas the corrupted signal is 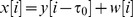 with 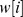 being a white contaminated Gaussian noise following the distribution of
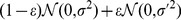
(52)where 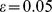 and 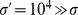. The time-delay 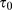 is set to be 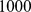 ms. Our purpose is to estimate 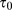 as accurate as possible under various signal to noise ratio 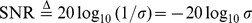. As illustrated in Figure 3, the procedure of estimating 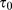 includes two steps. The first one is to construct a correlation function that corresponds to 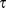 by each of 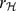, 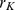 and 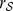 with respect to 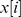 and 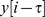. The second one is to locate time-shift 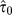 corresponding the maximum of the correlation function. The value of 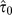 is considered to be an estimate of 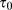 and restored for further analysis. Note that the number of Monte Carlo trials in this study is set to be 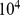.

Table 5 summarizes the estimates of 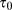 from 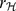, 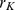 and 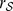. It is observed that all three methods produce acceptable estimates of 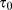, for an SNR even as low as 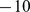 dB. However, PRVCC is slightly better than the other two, in the sense of giving smaller biases and standard deviations in most cases.

**Table 5:** Performance comparison of 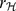, 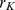 and 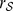 for 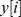 being a segment of sin wave.

SNR	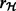	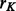	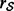
0	1000.0 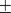 0.96	1000.0 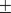 1.01	1000.0 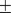 1.03
−1	1000.0 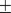 1.05	1000.0 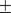 1.27	1000.0 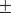 1.12
−2	1000.0 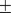 1.13	1000.0 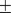 1.23	1000.0 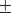 1.23
−3	1000.0 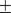 1.26	1000.0 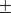 1.71	1000.0 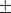 1.70
−4	1000.0 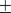 2.22	1000.0 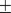 2.08	1000.0 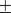 2.31
−5	1000.0 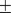 3.37	1000.0 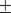 3.57	1000.0 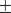 3.41
−6	1000.0 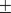 7.34	1000.1 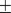 7.21	1000.0 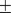 7.45
−7	1000.1 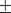 9.22	1000.1 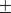 9.29	1000.1 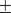 9.23
−8	999.9 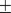 14.44	1000.2 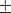 14.99	1000.1 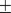 15.30
−9	1000.2 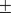 19.75	1000.2 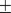 20.82	1000.1 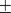 21.05
−10	999.7 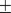 29.72	999.7 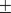 30.69	999.8 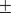 30.66

## Conclusions

This paper systematically investigates the statistical properties of the historical PRVCC under a particular contaminated Gaussian model. As shown in our previous work [20], this model simulates reasonably some frequently encountered scenarios where one variable is clean and the other corrupted by a tiny fraction of impulsive noise with very large variance. Under this model, we establish the asymptotic closed forms of the expectation and variance of PRVCC by means of the well known Delta method. To gain a further insight on PRVCC, we also compare it with two other classical correlation coefficients, i.e., SR and KT, in terms of the popular RMSE. Monte Carlo simulations not only verify our theoretical findings, but also reveal the strength and weakness of PRVCC in various occasions. The theoretical and empirical findings in this work are believed to add new knowledge to the area of correlation analysis that prevails in many branches of science and engineering.
